# Ultra-Fast Low Concentration Detection of *Candida* Pathogens Utilizing High Resolution Micropore Chips

**DOI:** 10.3390/s90301590

**Published:** 2009-03-09

**Authors:** Rafael Mulero, Dong Heun Lee, Michele A. Kutzler, Jeffrey M. Jacobson, Min Jun Kim

**Affiliations:** 1 Department of Mechanical Engineering & Mechanics, Drexel University, Philadelphia, PA 19104, U.S.A. E-Mail: rm63@drexel.edu; 2 Division of Infectious Diseases and HIV Medicine, Drexel University, Philadelphia, PA 19102, U.S.A. E-Mails: dlee@DrexelMed.edu; Mkutzler@DrexelMed.edu; Jeffrey.Jacobson@DrexelMed.edu;

**Keywords:** *Candida*, low concentration detection, ionic current blockage, micropore, pathogens

## Abstract

Although *Candida* species are the fourth most common cause of nosocomial blood stream infections in the United States, early diagnostic tools for invasive candidemia are lacking. Due to an increasing rate of candidemia, a new screening system is needed to detect the *Candida* species in a timely manner. Here we describe a novel method of detection using a solid-state micro-scale pore similar to the operational principles of a Coulter counter. With a steady electrolyte current flowing through the pore, measurements are taken of changes in the current corresponding to the shape of individual yeasts as they translocate or travel through the pore. The direct ultra-fast low concentration electrical addressing of *C. albicans* has established criteria for distinguishing individual yeast based on their structural properties, which may reduce the currently used methods’ complexity for both identification and quantification capabilities in mixed blood samples.

## 1. Introduction

*Candida* species are the fourth most common cause of nosocomial blood stream infections in the United States [1]. Early diagnostic tools for invasive candidemia are lacking, as current blood culture methods to detect *Candida* take up to 72 hours for conformation. Candidemia carries the highest associated mortality of all the blood stream infections [1]. Failure to initiate therapy in a timely manner is associated with poor clinical outcome (mortality). With no sensitive method of detecting candidemia, often time presumptive treatment (e.g., 2 weeks for fungemia) is used in high risk patients at the risk of drug resistance and drug toxicity [2]. The development of a more rapid detection and identification method for candidemia is needed. A rapid reliable test for candidemia might decrease hospital stay and the inappropriate use of antifungal treatments.

In most laboratories, detection of *Candida* from blood is commonly achieved by using standard bacterial blood culture media in an automated blood culture system (ABC)[3]. Culture media is inoculated at the bedside and placed in the ABC. Depending on the species, medium, and detection system, candidemia is detected within 24 to 72 hours [4]. Once detected, the *Candida* species is identified using commercial peptide nucleic acid fluorescent in situ hybridization (PNA-FISH) methodology. This process is able to identify common organisms such as *C. albicans* or *C. glabrata* in 3 hours. For final identification, *Candida* is subcultured and incubated for an additional 24 hours. The overall process takes 72 hours from the time the ABC detects a positive culture.

Prior clinical candidemia studies have evaluated the ability to detect candidemia using current blood culture methodologies, and have shown the occurrence of up to 35% false results [5]. Prolonged time was also needed to detect certain *Candida* species such as *C. glabrata* and *C. tropicalis* [4,5]. The reason for the high rate of false negatives is not well defined.

McMullan *et al.* [6] used a PCR method for early detection and identification of *Candida*. After manual extraction of DNA from serum, PCR was performed. This method shortens the procedure time up to 6 hours with similar accuracy outcome compared to conventional methods. However, processing requires multiple steps, and it may take longer to apply this method in clinical practice. With the increasing rate of candidemia, a new screening system is needed to detect the *Candida* pathogens in a timely manner.

**Figure 1.**
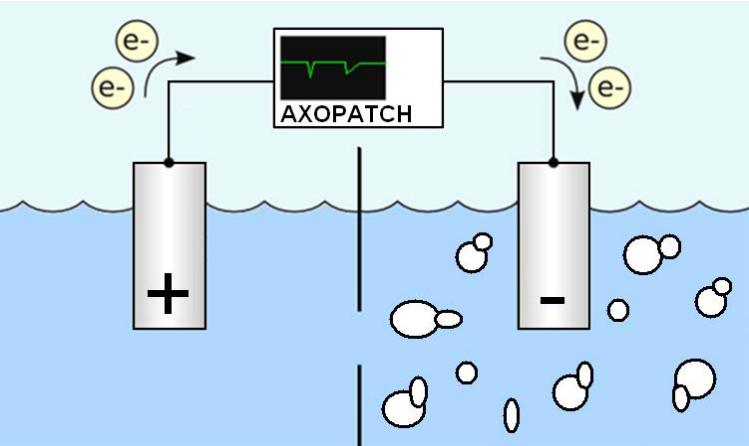
Conceptual illustration of electrolytic flow cell used to rapidly detect and configure yeast. The negatively charged yeast are drawn electrophoretically to the cathode, translocating through the micropore. This translocation causes an increase in the resistance to the ionic flow creating a signature temporary decrease in recorded current (ionic current blockade) relating to the size, configuration, and morphology of the yeast.

In this paper we present a novel method to identify yeast by their characteristic budded shape during mitosis by measuring the ionic current blockade (resistive pulse) [7] as the budded yeast electrophoretically translocate a micropore (Figure 1). While others have used long tubular Coulter counter devices to volumetrically size and count other types of biological particles [8–10], the resolution of their ionic current blockage signal for morphological detection is insufficient. Because of the relative thinness of the micropore substrate in comparison to the characteristic dimensions of the analyte we believe detection of not only the volume of micro-analytes is possible but also their sub-characteristic dimensional morphology. Low concentration samples of lab cultured Candida albicans were used in this study to demonstrate the efficacy of this method. Ten micrometer diameter pores where chosen to allow clearance of the ∼7 μm diameter yeast bodies and prevent clogging. High resolution electrical signal readout enabled the detection and discrimination of the orientation of both non-budded and budded yeast.

## 2. Methods and Materials

### 2.1. Micropore Fabrication and Flow Cell

**Figure 2.**
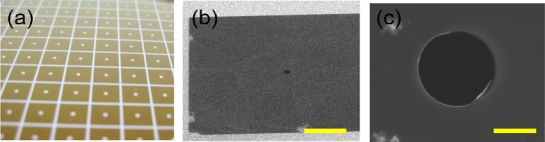
Micropore fabrication: (a) SiN wafer after photolithography and reactive ion etching processes, (b) SEM micrograph of free standing Si_x_N_y_ window after completion of 1.25 μm diameter pore taken at 5k×, 3kV with 52.0° tilt using ion beam on DB 235 focused ion beam (FIB) and (c) up close micrograph of 10 μm diameter pore used in experimentation. The scale bars are 7 μm and 5 μm in (b) and (c), respectively.

A prerequisite to making solid-state micropores is the ability to fabricate solid-state free-standing thin films. In this paper we use the following methods to fabricate our free standing thin film. Fabrication begins with the formation of a low-stressed silicon nitride membrane, 200 nm thick deposited across 340 μm thick silicon substrate wafer. SiN films can be deposited using low pressure chemical vapor deposition (LPCVD) at a temperature of 825 °C using ammonia and dichlorosilane gases [11–13]. The flow rate of ammonia to dichlorosilane is 1:5. This results in a silicon-rich nitride film, with a resulting tensile stress in the range of 50 to 150 MPa. A 50 × 50 μm^2^ window is then fabricated on the silicon substrate wafer using photolithography and standard KOH wet-etching [14,15]. Pores with diameters of 10 μm are directly milled in the thin film using a FEI Strata DB 235 FIB at a beam current of 50 pA and dwelling time of five minutes (Figure 2). Prior to experimental use the micropore chip is cleaned using Piranha solution, deionized water rinsed and dried carefully using an N_2_ stream.

**Figure 3.**
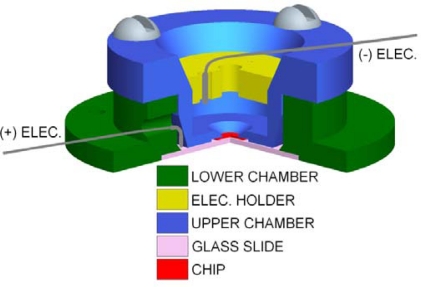
Rendering of experimental flow cell with a 90° radial cut-away for cross-sectional view. Upper chamber (blue), electrode supporting ring (yellow), low chamber (green), micropore chip (red), and glass slide (brown).

The flow cell (Figure 3) is made entirely out of polytetrafluoroethylene (PTFE) due to its low noise rating and chemically inert properties [16]. Prior to experimentation it is cleaned in RCA1 solution, rinsed with deionized water, and dried using an N_2_ stream before experimentation to prevent contamination. After cleaning, the micropore chip is adhered to the upper chamber using a fast curing silicone elastomer epoxy. Two 0.254 mm diameter PTFE coated silver electrodes with 1.5 mm exposed are then placed and sealed inside the upper and lower chamber. After curing, both chambers are filled with KCl and assembled using PTFE screws. The support ring defines the distance between the two electrodes and subsequently the electric field strength. The electrodes are connected to a Molecular Devices Axopatch 200B patch clamp amplifier which clamps a potential across the micropore while recording the resulting ionic current flow. The electrical data is sampled at 100∼250kHz, digitized using a MD Digidata 1440A digitizer, and analyzed using pClamp 10.1 software. To minimize any ambient electro-magnetic field (EMF) induced noise in the data signal the entire flow cell and patch clamp headstage are located inside a 5 mm thick copper Faraday cage.

### 2.2. Cell Preparation

**Figure 4.**
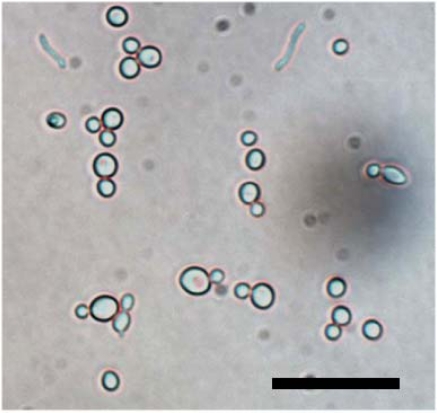
Phase contrast micrograph of a separated group of *Candida albicans* at saturated concentration. The scale bar is 25 μm.

*Candida albicans* (ATCC 14053, Figure 4) is cultured in agar plates. After culturing in the agar plate, colonies are diluted in 0.45 % normal saline. A 0.5 McFarland concentration of *Candida* suspension is prepared. It is further diluted in the desired concentration of 2 × 10^1^, 2 × 10^2^, 2 × 10^3^, 2 × 10^4^, and 2 × 10^5^ colony forming unit/mL. Each concentration is plated again to measure colony forming unit. Before experimentation solutions are agitated to disperse the organisms throughout the entire volume.

### 2.3. Micropore Characterization

**Figure 5.**
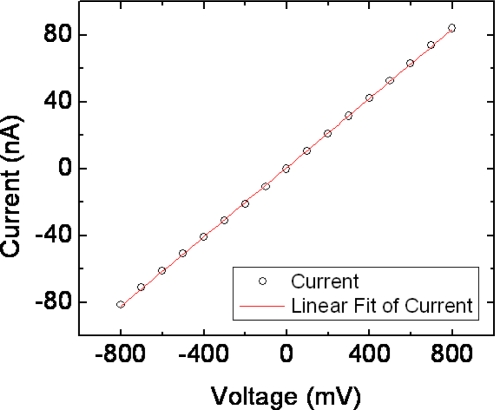
Current voltage characteristics of a 10 μm diameter solid state pore obtained at 0.001M KCl for the Candida albicans detection and configuration experiments. Curve demonstrates Ohmic I–V dependence. Its unobstructed conductance is found to be 100 nS. Error bars are too small to plot.

**Figure 6.**
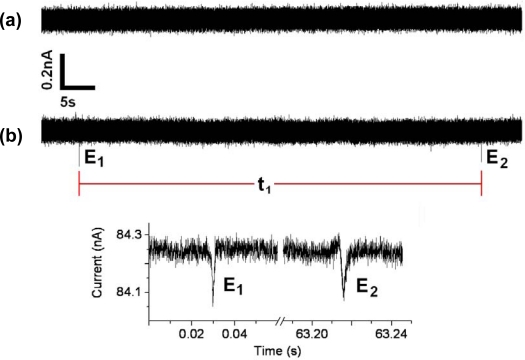
(a) Open conductance of a 10 μm pore at 700 mV applied potential. (b) Yeast translocation events using a 2 × 10^2^ yeasts/ml sample, caused by multiple translocations under a driving voltage of 1000 mV. Analyte concentration is characterized using time interval, *t_1_*. in between events *E_1_* and *E_2_*. (c) Detailed view of events *E_1_* and *E_2_*.

To find the conductivity of the pore an I–V curve is plotted measuring the current response to incremental increases in command voltage (Figure 5). The I–V plot’s linear slope is used to calculate its unobstructed conductance which in this case is found to be 100 nS. A plot of its steady-state ionic flow over time at a set command voltage is made for comparison to ionic flow later recorded during analyte sensing experimentation (Figure 6). After micropore characterization, the top chamber is evacuated and refilled with extremely low concentrations of isolated yeast suspended in the identical aqueous KCl solution concentration used for characterization.

## 3. Results and Discussion

By monitoring the conductance of a voltage biased pore, we detect translocation of *Candida* at a 1000 mV applied potential across a Ø10 μm solid-state pore. When the yeast is introduced into the pore electrophoretically, a resistive current pulse occurs reflecting the occupancy of the yeast cell within the pore. Translocation only occurs when the analyte dissociates, resulting in a characteristic spike (translocation event) to a low conductance state as the analyte moves through the flow limiting constriction.

Twelve minutes after the introduction of 750μl of 2 × 10^2^ CFU/ml yeast sample solution to the electrolytic cell the appearance of two translocation events occurred. This time delay until first detection should vary as it is a function of the initial location of the low concentration of yeasts when injected into the flow cell and their subsequent travel toward the sensing zone of the micro-pore. In this preliminary study we were able, however to detect our lowest concentration of *C. albicans* within minutes of sample insertion, compared to the 22 hours needed to detect *C. albicans* in the conventional automated blood culture system [3]. Additional translocation events were also recorded at higher concentration. The time interval between events (Figure 6) is proportionally related to the concentration of yeast in solution.

**Figure 7.**
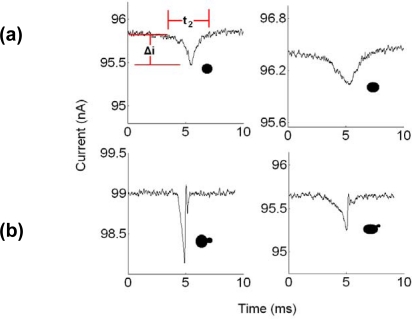
Typical current signatures observed. (a) Two examples of single yeast translocation events recorded using a 10 μm diameter pore. Events are characterized by their individual translocation times, *t_2_*, and current amplitudes, *Δi*. (b) Two examples of budding yeast cell translocations. The inset in both (a) and (b) is an illustration of the probable morphology of the translocating yeast cell.

The translocation events of *Candida albicans* have been analyzed. The amplitude, translocation time, and shape of the recorded translocation events vary with the conductivity of ionic solution, the electric field strength and distribution, the trajectory of yeast through pore, the amount of yeast within the pore, yeast/pore interaction, and the yeasts’ morphology, and orientation. During the discussed experiment the first two factors remained constant however the remaining factors clearly varied with individual translocations (Figure 7). Because of the relative thinness of the substrate in comparison to the characteristic dimensions of the analyte we believe detection of not only the volume of micro-scale analyte is possible but also their sub-characteristic dimensional morphology. Among the majority single resistive peaks observed, distinct pairs of resistive pulse peak amplitudes (Figure 7) corresponding to translocation of *C. albicans* into the micro-scale pore were also observed. This revealed the shape of a single cell yeast and budding yeast (blastodonidia) respectively, which were imaged in advance using phase contrast microscopy (Figure 4). The measurement of the translocation time and amplitude of the lower conductance state and characteristic budded peak shape allows the detection of *Candida* in a sample. Moreover, the time interval between two translocation events will indicate the concentration of *Candida* in the blood.

## 4. Conclusions

In this experiment, we demonstrate the feasibility of detecting yeast electrically using a micropore chip. We are able to detect the lowest concentration of *C. albicans* faster than the conventional automated blood culture system as well as characterize some of their physio-chemical properties. The characteristic budding morphology of *C. albicans* is atypical of the micro-scale material found in patient blood samples and as shown, maybe exploited for rapid detection. In order to achieve a clinically applicable technique pre-processing would be necessary to reduce or eliminate potentially pore clogging particles from sample such as red blood cells, white blood cells, and platelets. In addition to pre-processing, the solid-state pore’s composition allows for chemical, mechanical, or electrical modification which can be harnessed to develop pores which are able selectively translocate yeast while preventing interaction with material likely to cause clogging or hinder the pore’s sensing abilities. Future experiments involving tailored pore-analyte interaction through chemical modification of the pore [17] may also allow distinction between analyte and particles of similar size and morphology may be a critical feature in whole blood testing.
